# Amputee perception of prosthetic ankle stiffness during locomotion

**DOI:** 10.1186/s12984-018-0432-5

**Published:** 2018-11-08

**Authors:** Max K. Shepherd, Alejandro F. Azocar, Matthew J. Major, Elliott J. Rouse

**Affiliations:** 1 Shirley Ryan AbilityLab, Room 11-1414, 355 E Erie St, Chicago, IL 60611 USA; 2 Department of Biomedical Engineering, Northwestern University, 663 Clark St, Evanston, IL 60208 USA; 3 Neurobionics Lab, University of Michigan, Ann Arbor, MI 48109 USA; 4 Department of Mechanical Engineering, University of Michigan, Ann Arbor, MI 48109 USA; 5 Robotics Institute, University of Michigan, Ann Arbor, MI 48109 USA; 6 Northwestern University Feinberg School of Medicine, Department of Physical Medicine and Rehabilitation, 710 North Lake Shore Drive, #1022, Chicago, IL 60611 USA

**Keywords:** Prosthetics, Perception, Stiffness, Variable-stiffness

## Abstract

**Background:**

Prosthetic feet are spring-like, and their stiffness critically affects the wearer’s stability, comfort, and energetic cost of walking. Despite the importance of stiffness in ambulation, the prescription process often entails testing a limited number of prostheses, which may result in patients receiving a foot with suboptimal mechanics. To understand the resolution with which prostheses should be individually optimized, we sought to characterize below-knee prosthesis users’ psychophysical sensitivity to prosthesis stiffness.

**Methods:**

We used a novel variable-stiffness ankle prosthesis to measure the repeatability of user-selected preferred stiffness, and implemented a psychophysical experiment to characterize the just noticeable difference of stiffness during locomotion.

**Results:**

All eight subjects with below-knee amputation exhibited high repeatability in selecting their Preferred Stiffness (mean coefficient of variation: 14.2 ± 1.7%) and were able to correctly identify a 7.7 ± 1.3% change in ankle stiffness (with 75% accuracy).

**Conclusions:**

This high sensitivity suggests prosthetic foot stiffness should be tuned with a high degree of precision on an individual basis. These results also highlight the need for a pairing of new robotic prescription tools and mechanical characterizations of prosthetic feet.

## Background

Mobility is a key predictor of quality of life for lower-limb amputees [1]. Critical aspects of mobility, such as stability and energetic cost of walking, are highly affected by the mechanical behavior of leg prostheses [2–6]. The process of matching patients with appropriate prosthetic componentry is traditionally based on experiential decisions of clinicians. Consequently, selection of a specific component might be generally acceptable to a prosthesis user, but limit achievement of their full rehabilitation potential [7].

A common device prescribed to persons with lower limb loss is the modern energy storage and return (ESR) prosthetic foot, which intends to mimic the spring-like behavior of the biological ankle-foot complex during gait. ESR feet deflect during mid-stance, storing energy which is subsequently returned during terminal stance phase to help propel the body forward [8]. The effectiveness of springs in mimicking the function of the biological ankle is well supported by research on the quasi-stiffness and stiffness of the ankle during level-ground walking, as well as the time-tested success of spring-like commercial ankle-foot prostheses [9–11]. For ESR feet, stiffness—the ratio between applied force and associated deflection—is perhaps the most defining mechanical characteristic, and its effects on gait mechanics, metabolic cost of walking, socket comfort, and limb loading are well studied [2–6, 8].

Despite the importance of stiffness in locomotion, prosthetic manufacturers do not offer quantitative stiffness information—only qualitative scales associated with body weight and activity level—making cross-comparison between feet exceedingly difficult. The prescription process is further hindered by the excessive time required to test each prosthesis, as each tested device must be individually donned, aligned, adapted to, assessed, and doffed. As a result, it is not uncommon for patients to pilot only one or two prostheses; furthermore, the down-time required to switch prostheses hinders direct comparison of their impact on patients.

With the advent of computer-controlled active and quasi-passive / semi-active prostheses, new tools for improving the prescription process are on the horizon. Caputo et al. have proposed using a powered prosthesis emulator to adjust mechanical behavior of the prosthesis in real-time, thereby quickly simulating the mechanics of different prosthetic feet [12]. With such a device, the optimal foot behavior could be determined through various biomechanical objectives, such as minimizing metabolic cost or gait asymmetry. However, it is more likely that prosthetists and patients will work together, making fine adjustments to improve less-quantifiable metrics such as perceived levels of comfort, effort, and stability. With a patient-in-the-loop approach, prosthetists may adjust device parameters and ask patients for their preference, or patients may make the adjustments themselves [13]. However, the ability of patients to perceive and communicate changes in prosthesis properties—stiffness in particular—has not been quantified, despite being incorporated into the shared decision process of clinical fitting and alignment.

The resolution with which below-knee prosthesis users can discriminate changes in prosthesis stiffness has important implications for the balance of patient and clinician voices in the shared-decision prescription process. If, for instance, patients are highly sensitive to small changes in stiffness, and exhibit high repeatability in their preferences, then clinicians can rely more heavily on patient feedback. This could be particularly true if patient sensitivity to stiffness proves to be of substantially higher resolution than clinicians’ perception of the corresponding gait changes. If, conversely, patients exhibit poor sensitivity, or have difficulty finding or communicating their preference, then prosthetists may need to emphasize alternative metrics for assessment.

In this experiment, we sought to determine the sensitivity of below-knee prosthesis users to prosthetic ankle stiffness. Specifically, we assessed 1) the repeatability (variability) of user-selected preferred stiffness, and 2) the difference threshold, or Just Noticeable Difference (JND), for stiffness during locomotion. Additionally, we sought to determine if simple measures, such as self-reported mobility assessment and peripheral vibration sensing, accurately predicted perception of stiffness. Our results provide a rigorous yet patient-centric perspective on the importance of optimizing stiffness on an individual basis, and provide valuable insight into the potential for new prescription methods using robotic devices.

## Methods

### Overview

To perform this experiment, we used a custom Variable Stiffness Prosthetic Ankle-Foot (VSPA Foot), which has the distinct advantages of providing known torque-angle mechanics, while enabling adjustment of stiffness in real-time, between steps during locomotion [14]. First, subjects walked on a treadmill while wearing the VSPA Foot, and used a dial to repeatedly select their preferred stiffness. We then quantified their ability to perceive changes in stiffness by making small changes to stiffness and asking whether the patient could identify if the stiffness increased or decreased. A psychometric curve was then fit to these data to determine subjects’ difference threshold of stiffness perception. Preliminary results from a portion of this study were presented at the 2018 International Conference on Biomedical Robotics and Biomechatronics.

### Variable-stiffness ankle-foot prosthesis

**Fig. 1:**
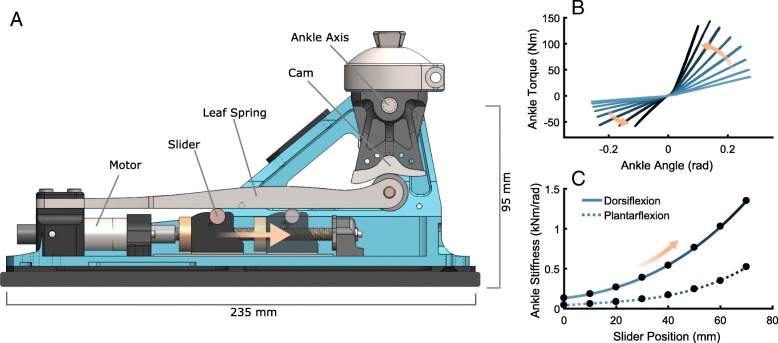
**a** Modified version of the VSPA Foot [14]. As the slider is actively repositioned towards the cam, the stiffness increases (orange arrows). The cam was designed to create constant stiffness (a linear torque-angle relationship). **b** Experimental characterization of select torque vs. angle curves across the range of slider positions. **c** Ankle stiffness, defined as a function of slider position with a cubic fit. Dorsiflexion stiffness (filled line) is considered the nominal stiffness for this study

The VSPA Foot (modified from Shepherd and Rouse, 2017 [14]) was used to perform this study (Fig. 1a). The VSPA Foot can change its stiffness by over an order of magnitude, mimicking the mechanical behavior of a range of ESR feet.^1^ It employs an actively repositionable simple support beneath a leaf spring to change stiffness, and a cam-based transmission to create a custom torque-angle curve (Fig. 1b). Modifications to the VSPA Foot design included a stiffer aluminum chassis and inclusion of a 6AL-4 V titanium spring, rather than the fiberglass spring implemented in the original design; both of these modifications were implemented to increase the maximum attainable stiffness of the foot. In addition, the cam-follower was integrated into the titanium spring design, which enabled a more symmetric change to the dorsiflexion and plantarflexion mechanics as foot stiffness was increased or decreased.

The cam profile was designed to create a linear torque-angle relationship at the ankle joint in both plantarflexion and dorsiflexion, providing the mechanics of a simple torsional spring at the ankle joint. Plantarflexion stiffness (the stiffness when the ankle is plantarflexed from neutral, occurring immediately following heel strike) was designed to be 33% of dorsiflexion stiffness. This ratio follows work completed by Major et al. [2] based on characterization of common ESR feet, and from subject feedback during pilot testing.

The achievable stiffness levels were measured using a custom rotary dynamometer and an onboard encoder on the ankle axis (Fig. 1b). Hysteresis is minimal; less than 5% of energy stored is lost. For all tests, when describing stiffness quantitatively, we refer only to *dorsiflexion* stiffness, as the ankle is primarily spent dorsiflexed from neutral during stance phase. The relationship between dorsiflexion stiffness and slider position is described by a cubic fit to the characterization data (Fig. 1c).

The ankle could be commanded to change stiffness by either the experimenter, who could communicate with the onboard computer (*Raspberry Pi Zero*, Raspberry Pi Foundation, Cambridgeshire UK) over WiFi, or directly by the subject, using a small dial which could rotate indefinitely and had no absolute reference. The ankle was worn without a shoe to avoid additional compliance and confounding factors [15]. Instead, a 6 mm piece of shoe material (*SolFlex crepe*, Shore A durometer 50–55, SoleTech, Nahant, MA, US) was epoxied to the bottom of the foot to aid in shock absorption, and a thin tread (*Diamond*, durometer 50–55, SoleTech) was epoxied below that shoe material for traction.

### Participants

Eight unilateral below-knee amputees were recruited to participate in the study (Table 1). Participants were at least nine months post-amputation, able to walk one km on a treadmill over the course of 45 min, 18–65 years old, 60–95 kg, and primarily used an ESR or hydraulic foot at home. None of the subjects had skin irritation on the affected leg or comorbidities that would affect their gait biomechanics. Informed consent was obtained by each subject prior to testing, and the study was approved by the Northwestern University Institutional Review Board.

**Table 1:** Subject Information

Subject	Age (yrs)	Height (m)	Weight (kg)	Time since amputation (yrs)	Residual limb length (cm)	Customary prosthesis	Amputation etiology	PLUS-M raw score (T-Score)
1	23	1.88	86.2	3	14	Össur *Vari-Flex XC*	Traumatic	60 (71.4)
2	41	1.52	76.2	4	15	Freedom Inn. *Senator*	Dysvascular	58 (64.5)
3	41	1.70	54.4	14	12	Össur *Elation*	Traumatic	43 (49.1)
4	46	1.85	86.0	26	24	College Park *Velocity*	Traumatic	60 (71.4)
5	33	1.75	72.5	13	15	College Park *Soleus*	Traumatic	36 (44.5)
6	24	1.65	61.2	1	14	Össur *Pro-Flex LP*	Traumatic	54 (58.4)
7	35	1.83	90.0	15	14	Ability Dyn. *Rush Foot*	Dysvascular	53 (57.3)
8	54	1.78	84.0	1	15	Endolite *Echelon*	Dysvascular	54 (58.4)

Prior to the walking experiments, anthropometric data were taken, and several tests were administered for possible correlations with stiffness perception. Each subject’s height, weight, side of amputation, take-home prosthesis type, and length of affected limb (mid-patellar tendon to distal end) were recorded (Table 1). Peripheral vibration threshold was assessed with a Rydel-Seiffer Tuning Fork (US Neurologicals, LLC, Poulsbo, Washington US) on three bony prominences with three trials per location: amputation-side tibial tuberosity, sound-side tibial tuberosity, and sound-side medial malleolus. All trials occurred with the subject seated with eyes closed, and leg extended, resting on a stool. The scores for the three locations were averaged to create an overall vibration-sensing score. Finally, the Prosthetic Limb Users Survey of Mobility (PLUS-M) self-report instrument was administered, with the maximum score of 60 and higher scores suggestive of higher mobility [16].

### Fitting and familiarization

Subjects first walked on the treadmill with their customary prosthesis for several minutes to familiarize themselves with the treadmill and laboratory space. The customary prosthesis was then removed, and the VSPA Foot was fit and aligned to their customary socket by a certified prosthetist. The subjects then walked on the treadmill with the VSPA Foot set to an initial stiffness given by *k* = 7.5 x Mass (kg), a standardized setting established from preliminary experiments. The prosthetist stopped the treadmill as necessary to adjust the alignment. During this process, the speed of the treadmill was slowly increased by the subject to 1.0 m/s, which was the nominal experiment speed. All experiments were performed at 1.0 m/s, except by Subjects 2, 4, and 6, who felt uncomfortable at this speed and elected to walk at 0.87 m/s. This speed is slightly less than is typically reported for self-selected walking speed, but was selected to ensure stability during trials in which the stiffness was changed between steps. When the prosthetist and subject were content with the alignment, and the subject expressed comfort walking with the prosthesis at the experiment speed, the treadmill was stopped for a short break before Experiment 1.

### Experiment 1: Preferred stiffness

After familiarization and alignment, the subjects walked on the treadmill and were asked to “select the stiffness that you find to be the most comfortable at this walking speed.” To change foot stiffness, subjects were free to rotate the dial at any time during the gait cycle; however, active stiffness adjustments were made only during the swing phase of gait. Subjects were encouraged to explore a range of stiffness levels until the prosthesis felt uncomfortably stiff or uncomfortably soft. After selecting their preferred stiffness (PS), the treadmill was stopped, and the researcher “reseeded” the stiffness by pseudo-randomly increasing or decreasing it by 25%. The subjects repeated the process of selecting a PS for a total of five trials. The process typically took longer (approximately 10 min) for the first trial, and less time in subsequent trials, as the subjects learned the effects of changing stiffness and became more confident in their choices. Subject 2 did not fully understand the instructions and had to be retested on a different day.^2^ We characterized each subject’s repeatability of selecting their PS with Coefficient of Variation (CV_PS_), which is the ratio of the standard deviation (unbiased estimation) to the mean.

### Experiment 2: Stiffness perception

Stiffness perception was assessed on the same day as Experiment 1, following a short break. Stiffness perception was determined by changing the stiffness of the prosthesis while subjects walked on the treadmill; specifically, we implemented a two-interval forced-choice (2IFC) task and the method of constant stimuli [17, 18]. Subjects were presented with sequential pairs of stiffness values and asked whether the second stimulus was “stiffer” or “less stiff” than the first. Each pair of stimuli constituted a single trial, and consisted of a *reference* stiffness and a *comparison* stiffness. The reference stiffness corresponded to each subject’s mean preferred stiffness (PS) and was constant across all trials. The comparison stiffness varied between trials, and was equal to the PS scaled by a value in the set: {0.80, 0.85, 0.90, 0.95, 1.0, 1.05, 1.10, 1.15, 1.20}. This range was informed by preliminary experiments, with the intent of generating both incorrect answers for the smallest changes and very high accuracy for the largest changes. Each comparison stiffness was presented eight times, for a total of 72 trials. The reference stiffness was presented first on half of the trials, and second on the other half. The order of stiffness values was set before the experiment. The trials were pseudo-randomized; to avoid destabilizing the subjects, the trials were randomly re-shuffled until all consecutive stiffness values were within 25% of each other.

Prosthesis stiffness was changed by the experimenter approximately every 6–8 strides, and the change was indicated to subjects by an audible tone. The number of strides was chosen to allow subjects ample time to sense and report the stiffness changes, while minimizing the amount of time spent walking. Subjects only responded after the transition from the first stimulus to the second stimulus (i.e., they did not respond when stiffness was changed between trials). Subjects were allowed to take breaks whenever necessary to avoid fatigue bias. Before the official trials began, subjects were trained with stiffness changes of 15–25%, until subjects reported that they clearly understood the paradigm.

For each stiffness value, the proportion (*P*) of trials judged stiffer than the reference was used to calculate the psychometric function, a sigmoidal function which describes the relationship between stiffness change and probability of perceiving that change as an increase in stiffness. Specifically, a logistic function was fit using a maximum likelihood criterion, with lapse rate fixed at 0.02. The lapse rate accounts for trials in which subjects respond independently of stimulus level (e.g., due to a lapse of attention), and reduces biases on the parameters that determine the psychometric function [17]. The comparison stiffness levels corresponding to *P* = 0.25 and *P* = 0.75, denoted *X*_*0.25*_ and *X*_*0.75*_, are used to calculate the threshold of perception. This threshold is commonly termed the Just Noticeable Difference (JND), and is specifically calculated as:

$$ JND=\frac{X_{0.75}-{X}_{0.25}}{2} $$

In this study, the JND represents the smallest *percent* change in stiffness that can reliably (with 75% accuracy) be identified as having been an increase or decrease in stiffness.^3^ Lower JND values correspond to better perception, since a smaller change in stiffness can be perceived.

At the end of the experiment, subjects were asked to describe the strategies they used to sense changes in stiffness. As mentioned previously, Subject 2 did not originally understand the instructions for selecting preferred stiffness, so their JND trials were performed with a reference stiffness of 571.5 Nm/rad (weight normalized: 7.5 Nm/rad/kg) and preferred stiffness was reassessed on a second day.

### Kinematics

To assess the sensitivity of ankle kinematics to ankle stiffness, and specifically the magnitude of biomechanical changes associated with the JND, ankle kinematic data were recorded in separate walking trials from the onboard ankle encoder. Subjects walked on the treadmill for 90 s at each of five stiffness levels (order randomized): the mean preferred stiffness modified by 0%, ± 10%, and ± 20%.

The kinematic data were recorded at 30 Hz by an onboard 14-bit absolute encoder, zero-lag filtered with a fourth-order, 7.5-Hz Butterworth filter in post-processing, and then up-sampled to 100 Hz with a spline interpolation. An angle of zero corresponded with the average angle during swing. The last 20 of each subject’s strides were segmented and averaged. The kinematics were then averaged across subjects at each of the tested stiffness levels. An estimate of the kinematics at the preferred stiffness ± JND was created with a linear interpolation from the ankle kinematics at the closest measured stiffness levels.

### Analysis

Each fitted psychometric function was ensured to have an acceptable goodness of fit [17, 19]. Means and standard errors were calculated across participants for both JND and CV_PS_. Neither JND nor CV_PS_ were normally distributed (*p* < 0.05, one sample K-S test), therefore standard errors were calculated with bootstrapping (*n* = 10,000 resamples, s.e.m. calculated as standard deviation of the bootstrapped means).

To test whether there was a relationship between stiffness perception and consistency of preferred stiffness selection, individual JNDs were linearly regressed on CV_PS_. JNDs were also regressed on PLUS-M T-scores and Vibration sensing score (averaged scores from the three tested anatomical locations) to see if either simple test may be used to predict perception.

## Results

### Experiment 1: Preferred stiffness

**Fig. 2:**
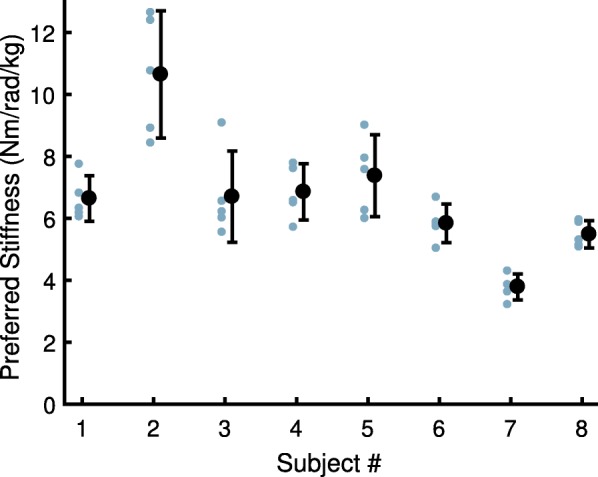
Preferred stiffness for each subject. Individual trials are shown next to each subject’s mean (error bars: SD)

Subjects’ self-selected preferred stiffness (Fig. 2) ranged from 3.8 to 10.7 Nm/rad/kg (or 341.1 to 811.4 Nm/rad,not weight-normalized). The mean weight-normalized preferred stiffness was 6.7 ± 2.0 Nm/rad/kg (mean ± s.d.). The mean within-subjects coefficient of variability (CV_PS_), which serves as a measure of the subjects’ consistency of selection, was 14.2 ± 1.7%.

### Experiment 2: Threshold of stiffness perception

**Fig. 3:**
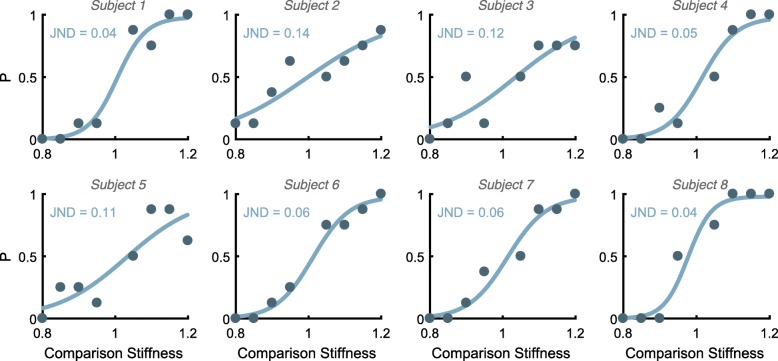
Individual psychometric curves and JNDs. The Comparison Stiffness (x-axis) is a fraction of each subject’s preferred stiffness (the reference stiffness), and the y-axis label *P* represents the proportion of trials judged stiffer than the reference

Psychometric functions were fit to the data for all eight subjects (Fig. 3), and individual JNDs ranged from 3.7 to 13.6%. The mean JND was 7.7 ± 1.3%.

**Fig. 4:**
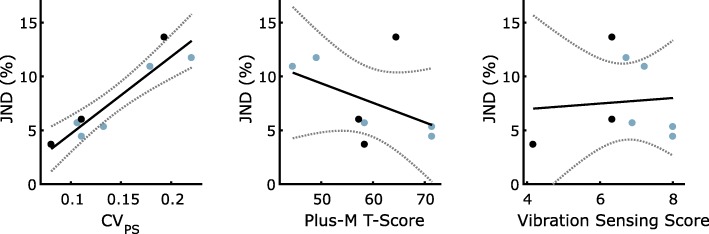
(Top) Just Noticeable Difference (JND) vs. Preferred Stiffness Variability. (Middle) JND vs. Prosthesis Limb Users Survey of Mobility (PLUS-M) T-Score. (Bottom) JND vs. Vibration Sensing Score; higher score indicates higher sensitivity to vibration. The three darker points denote the three dysvascular subjects

The individual JNDs were regressed against three predictors: preferred stiffness variability (CV_PS_), PLUS-M score, and vibration sensing score (Fig. 4). There was a significant (*p* < 0.001) positive correlation between CV_PS_ and JND. There was a non-significant (*p* = 0.25) negative trend between self-reported mobility (PLUS-M score) and JND; removing Subject 2 due to acquiescence bias (see endnote 2), this correlation became significant (*p* = 0.04). As expected, the dysvascular subjects tended to have worse vibration sensing, but there was not a significant correlation between vibration sensing score and JND (*p* = 0.85).

### Kinematics

**Fig. 5:**
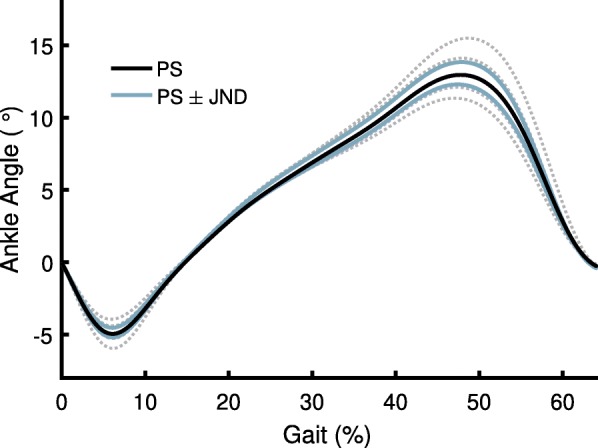
Pooled ankle kinematics of stance phase at five tested conditions: PS (black line), PS ± 10% and ± 20% (dotted gray lines). Also shown are estimates of the ankle kinematics at the PS ± JND (blue lines), which are linearly interpolated from the nearest measured stiffness values. Increasing range of motion corresponds with decreasing stiffness

For all subjects, lower stiffness levels resulted in increased ankle range of motion (Fig. 5). An estimate of the kinematics at the JND above and below the PS is shown in blue; the change in peak dorsiflexion associated with the JND was 0.77°. The kinematics are not representative of those seen during Experiment 2, as the short time spent at each level did not allow subjects adequate time to adapt their gait. They are instead presented to demonstrate the magnitude of kinematic changes during steady-state gait associated with the threshold of stiffness perception.

## Discussion

The purpose of this study was to determine the sensitivity of below-knee prosthesis users to changes in prosthetic ankle stiffness during walking by measuring the repeatability of user-selected preferred stiffness and difference threshold for stiffness. The mean JND of 7.7% is smaller than for some other physical tasks, including able-bodied subjects’ ability to sense the stiffness of a spring placed in parallel with their ankle (11.6%, [20]) or elbow (16%, [21]) while in a seated position. Stiffness sensing in these studies relied on integrating the sensations of torque and angle, which are likely dominated by feedback from local mechanoreceptors. Stiffness changes during ambulation, in contrast, may cause global kinematic and spatial orientation changes that can be sensed by the vestibular system. Specifically, at low stiffnesses, most subjects described the forward propulsion as inadequate, or the feeling of drop-off effect after entering midstance. Some subjects reported feeling like the stiffer setting made them stand taller during stance phase. Two subjects indicated feeling increased pressure between their knee and socket, but only at the highest stiffness levels.

There was large between-subject variability in JND, which was highly correlated with the repeatability of preferred stiffness selection (CV_PS_). Specifically, subjects with high repeatability in selecting preferred stiffness could perceive smaller changes in stiffness, suggesting that the ability to consistently select a preferred stiffness may depend largely on the ability to sense it (Fig. 4). As expected, vibration sensing threshold was worse in the three dysvascular subjects, but our results suggest that sensitivity to prosthetic ankle stiffness may not be dependent on residuum vibration sensation. Further research should consider other sensory factors that may contribute to prosthesis stiffness detection.

### Implications for prescription

Our results suggest that patients’ ability to effectively communicate stiffness preference is a critical piece missing in the current prescription process. The relatively low CV_PS_ of 14.2% provides a frame of reference for the degree of specificity expected in patient preference. Similarly, the between-subjects variability in weight-normalized PS highlights the need to pilot more than the manufacturer-recommended feet, which are based on weight and activity level, and which would have been almost the same for all eight subjects.

Whether or not a patient’s preferred stiffness is close to the ‘ideal’ stiffness is unknown. There are a multitude of quantitative and qualitative factors that are likely to be optimized in combination by either the prosthetist or patient, such as step length symmetry, limb loading, roll-over, or socket comfort [7]. Moreover, what a patient prefers in a clinical setting may not be the most comfortable, stable, or energetically optimal outside the clinic or long-term; adaptation of gait can continue for weeks past prescription [22]. Nevertheless, our results point to a need to more formally incorporate patient feedback of stiffness into the prescription process.

There are several limitations in the current prescription process that hinder the ability for subjects to sense and report preference for stiffness. First, it is challenging to efficiently test a range of commercial prosthetic feet in the clinic. The excessive time required to change feet, variability in alignment, and subtle differences in mechanical behavior make direct comparison of feet difficult. As a result, patients are unlikely to develop an intuitive sense of stiffness as a modifiable variable. We found that when introduced to stiffness as a controllable variable which could be adjusted instantaneously and continuously, most subjects internalized its effects, and settled on a small range of stiffnesses that they found to be most comfortable.

An approach to prescription based on systematically optimizing foot mechanics and alignment could only be feasible using robotic tools. Prosthetists and patients could work together to systematically and efficiently explore a set of mechanical variables, such as stiffness and alignment. This approach has been advocated by Caputo et al., who developed a tethered robotic platform which can emulate the mechanics of various prosthetic feet, both passive and active [12, 13].

For the described clinical optimization process to be effective, it would also have to be paired with standardized characterization of prosthetic foot behavior, which is not routinely provided by manufacturers [23]. Currently, prosthetists will either use their experience to guide them as to which feet might have a more appropriate stiffness, or make small adjustments to alignment, which has some effects similar to varying stiffness [23]. This relationship has not been studied directly, but Hansen has suggested that for feet with different mechanics, prosthetists change alignment to conform to an ideal roll-over shape [24]. Adjusting alignment is not a perfect substitute to changing ankle stiffness; while both affect the radius of curvature of the effective rocker, other roll-over characteristics vary substantially with alignment, such as arc length and horizontal displacement of the center of curvature [25].

### Implications for research

The high sensitivity of prosthesis users characterized here also has implications for how stiffness should be used as a manipulated variable in research. Several researchers have studied the effects of prosthetic ankle-foot stiffness on metrics such as stability, metabolic cost, and muscle activity. Researchers have often attempted to match the tested stiffness levels to that of common prostheses. However, the chosen variation in stiffness—which typically consists of only two or three discrete levels—has spanned a factor of 2.0 [26], 2.25 [27], 3.5 [6], and 6.2 [3]. The results of our study—both the repeatability of preferred stiffness and the sensitivity to stiffness changes—suggest that researchers may be testing unrealistic ranges of stiffness, and future studies should test a narrower window of stiffness, centered around subjects’ preferred stiffness.

This experiment was enabled by the highly predictable, repeatable, and accurate behavior of the VSPA Foot as a research tool. It is unlikely that this experiment would have been possible with a fully-powered prosthesis controlled to emulate springs, as these devices typically have noise levels in their torque control approaching or exceeding the lowest JNDs measured in our experiment [12, 28]. With 0.1-mm positioning accuracy for the stiffness-varying mechanics, the VSPA Foot can make very subtle changes, and because the behavior is intrinsically passive, the motion is guaranteed to be repeatable and noise-free. Researchers seeking to answer similar questions should consider using similar quasi-passive devices that can actively modify their intrinsically passive mechanics, to mitigate the confounding effects of torque-tracking error in fully powered devices.

### Limitations

A critical element of this study was training the subjects to conceptualize “stiffer” vs. “softer/less stiff.” Most subjects easily internalized the concept when they turned the dial and immediately felt the stiffness change. Both Subject #3 and Subject #5 had challenges with short-term memory loss due to the nature of their pathology, and Subject #3 requested re-training twice during the JND portion of the experiment. It seems evident that, in a clinical setting, some patients would be able to quickly grasp the concept and provide feedback of their preferences, whereas others may need more training. Similarly, the results of Experiment 2 (specifically, the ability to directionally identify changes in stiffness) were likely dependent on learning the sensations and effects of stiffness during the exploration in Experiment 1.

The pool of subjects is not a representative sample of all lower-limb amputees, due to the inclusion criteria (specifically the 90 kg upper weight limit and ability to walk for 45 min). It is possible that lower-level ambulators (below the Medicare Classification Level K3) will be less sensitive to stiffness, due to a variety of factors such as less experience walking with a prosthesis, or comorbidities such as peripheral neuropathy or excessive adipose tissue in the residuum.

Preferred stiffness is likely dependent on several factors which were controlled in our experiment, but may vary in a clinical setting. It is unknown how changes to alignment affect preferred stiffness; this is a potential avenue for future study. Similarly, we elected not to use a shoe on the prosthetic foot to facilitate complete control of stiffness adjustments, but shoes can add substantial series compliance [15], which would likely affect both preferred stiffness and JND. Stiffness preference was assessed at a steady speed on a treadmill, and may differ during over-ground walking with frequent stops. Finally, foot length may affect preferred stiffness, and may have a substantial effect on the kinematics [29].

Due to the mechanics of the VSPA-Foot, prosthesis plantarflexion and dorsiflexion stiffness could not be changed independently. We made the assumption that subjects would be most sensitive to dorsiflexion stiffness, and thus deemed dorsiflexion stiffness the nominal stiffness in the experiments. Feedback from subjects regarding their strategy confirmed this, but it is possible that we would have received different results with independent variation in stiffness or a different ratio.

## Conclusions

Below-knee amputees were able to sense a 7.7% difference in prosthetic ankle stiffness, and demonstrated high repeatability in selecting their preferred stiffness (coefficient of variability of 14.2%). This information substantiates the argument that that new tools are needed to allow patients and prosthetists to quickly locate an optimal stiffness based on shared decisions, and that standardized systems for measuring and reporting prosthesis stiffness should be adopted by manufacturers, researchers, and clinicians. Additionally, these results should inform future studies investigating the effects of prosthesis stiffness, providing a weight-normalized reference stiffness and a feasible range of stiffness levels to test.

## Abbreviations

ESR: Energy storage and return
JND: Just noticeable difference
PLUS-M: Prosthetic limb users survey of mobility
PS: Preferred stiffness
VSPA Foot: Variable-stiffness prosthetic ankle-foot

## Footnotes

1 While the exact mechanics of ESR prosthetic feet are complex, a common, simplified model consists of a solid foot attached to the shank via a rotational spring, articulating around an estimated ankle axis. Damping is present to varying degrees, but elasticity (stiffness) dominates the mechanics; for example, in ESR feet without a shock absorber (i.e., no damping), only 5–15% of the energy stored is lost [30].

2 We believe Subject 2 exhibited acquiescence bias, in which they were biased to report what they thought the experimenters wanted to hear. Specifically, during the preferred stiffness test, they turned the knob a small amount, without exploring the range, and claimed that the stiffness was their favorite. Accordingly, their results depended almost entirely on the random reseeding of stiffness between trials. Similarly, we believe their self-assessment of mobility in the Plus-M score was highly biased to show high activity level, which is in contradiction to their conversational anecdotes regarding their mobility, and our prosthetists’ assessments. We asked them to return on a second day to redo the preferred stiffness test, and made it clear that we wanted them to explore the range of stiffness levels (until it was uncomfortable) before selecting their preferred stiffness for each trial.

3 The JND is traditionally in the same units as the stimulus (e.g., Nm/rad), and is then normalized by the average stimulus intensity to calculate the Weber Fraction. However, since every subject had a different reference stiffness (their preferred stiffness), we chose to test their sensitivity as a *percentage* of their preferred stiffness (as opposed to our comparison stimuli being, for example, ± 20 Nm/rad for all subjects). Thus, in this experiment, the JND is equivalent to the Weber Fraction.
